# Nitric Oxide Mediates Crosstalk between Interleukin 1β and WNT Signaling in Primary Human Chondrocytes by Reducing DKK1 and FRZB Expression

**DOI:** 10.3390/ijms18112491

**Published:** 2017-11-22

**Authors:** Leilei Zhong, Stefano Schivo, Xiaobin Huang, Jeroen Leijten, Marcel Karperien, Janine N. Post

**Affiliations:** 1 Developmental BioEngineering, MIRA Institute for Biomedical Technology and Technical Medicine, University of Twente, 7522 NB Enschede, The Netherlands; zhongleilei8@gmail.com (L.Z.); s.schivo@utwente.nl (S.S.); x.huang-1@utwente.nl (X.H.); j.c.h.leijten@utwente.nl (J.L.); h.b.j.karperien@utwente.nl (M.K.); 2 Department of Orthopaedic Surgery, University of Pennsylvania, Philadelphia, PA 19104, USA; 3 Formal Methods and Tools, CTIT, University of Twente, 7522 NB Enschede, The Netherlands

**Keywords:** osteoarthritis, cell signaling, IL1β, WNT, antagonists, computational modeling, nitric oxide

## Abstract

Interleukin 1 beta (IL1β) and Wingless-Type MMTV Integration Site Family (WNT) signaling are major players in Osteoarthritis (OA) pathogenesis. Despite having a large functional overlap in OA onset and development, the mechanism of IL1β and WNT crosstalk has remained largely unknown. In this study, we have used a combination of computational modeling and molecular biology to reveal direct or indirect crosstalk between these pathways. Specifically, we revealed a mechanism by which IL1β upregulates WNT signaling via downregulating WNT antagonists, DKK1 and FRZB. In human chondrocytes, IL1β decreased the expression of Dickkopf-1 (DKK1) and Frizzled related protein (FRZB) through upregulation of nitric oxide synthase (iNOS), thereby activating the transcription of WNT target genes. This effect could be reversed by iNOS inhibitor 1400W, which restored DKK1 and FRZB expression and their inhibitory effect on WNT signaling. In addition, 1400W also inhibited both the matrix metalloproteinase (MMP) expression and cytokine-induced apoptosis. We concluded that iNOS/NO play a pivotal role in the inflammatory response of human OA through indirect upregulation of WNT signaling. Blocking NO production may inhibit the loss of the articular phenotype in OA by preventing downregulation of the expression of DKK1 and FRZB.

## 1. Introduction

Osteoarthritis (OA) is the most common joint disorder with the knee being the most affected joint. Knee OA affects >10% of the western population over 60 years of age, and this number is likely to increase due to the aging and obesity of the population [1]. OA affects the whole joint and as yet there is no cure. OA is characterized by progressive degeneration of articular cartilage, mild signs of inflammation, and typical bone changes [2,3]. The mechanisms underlying OA pathogenesis are still largely unknown.

Accumulating evidence has strongly linked WNT activity to the onset and development of OA. Indeed, alterations of WNTs and WNT-related proteins, such as the WNT antagonists Dickkopf-1 (DKK1) and Frizzled related protein (FRZB) have been found in human OA. Multiple whole genome studies indicated that loss-of-function single nucleotide polymorphisms (SNPs) in the WNT antagonist FRZB are related with hip OA [4,5]. In addition, FRZB-knockout mice have more severe OA cartilage deterioration in response to instability, enzymatic injury, or inflammation [6]. Moreover, FRZB^−/−^ mice are shown to have increased MMP expression after load or interleukin 1β (IL1β) treatment [7]. It was shown that high levels of DKK1 have a protective function against cartilage degeneration and that lower levels of DKK1 are associated with OA development [8,9,10]. We have previously shown that the exogenous addition of high concentrations of DKK1 and FRZB prevented the hypertrophic differentiation of chondrogenically differentiating mesenchymal stem cells [11]. In addition, we reported the loss of DKK1 and FRZB expression in OA [12], and showed that the expression of these antagonists are negatively correlated with grading of knee OA [13].

Interleukin 1β (IL1β) is a key pro-inflammatory cytokine that drives OA progression by inducing the expression of cartilage degrading enzymes, such as matrix metalloproteinases (MMPs) [14,15]. Pro-inflammatory cytokines stimulate iNOS (nitric oxide synthase) expression resulting in the synthesis and release of nitric oxide (NO), which contributes to the joint pathology [16,17]. NO is highly expressed in OA chondrocytes [18,19,20] and cartilage [21]. NO inhibits both the synthesis of proteoglycan and collagen [22], activates MMPs, mediates chondrocyte apoptosis [23], and promotes inflammatory responses. All of these effects contribute to the catabolic activities of NO in cartilage [24].

Despite the important roles of WNT and IL1β signaling in OA, it remains largely unknown how these pathways cross communicate and thereby affect OA onset and development. We have shown that WNT/β-catenin inhibits IL1β induced MMP expression in human articular cartilage. Addition of IL1β to human chondrocytes increased expression of WNT7b, while decreasing the expression of the WNT antagonists DKK1 and FRZB. This correlated with an increase in β-catenin accumulation [25]. In addition, the WNT/β-catenin regulated transcription factor TCF4 (Transcription Factor 4) binds to NF-κB (Nuclear Factor κB) thereby enhancing NF-κB activity [26].

It was recently shown that IL1β induced NO production in cancer cells was responsible for a strong decrease in DKK1 expression, which in turn resulted in the upregulation of WNT/β-catenin signaling [27]. However, the mechanism by which IL1β downregulates DKK1 and FRZB in chondrocytes is as yet unknown and the subject of this manuscript.

Since OA is a complex disease, involving integration of many factors leading to a unique response in cell fate, a thorough understanding of the integration of signals in cells and disease pathologies is necessary for the development of effective therapies [28]. In the past, attempted clinical trials relied on the correlation of a single pathway. However, as yet there are no successful treatment strategies that successfully treat OA and prevent cartilage degeneration (reviewed in [29]). Static diagrams of signal transduction pathways prevent insight into the dynamic behavior of these systems. Pathways are often studied in isolation, largely deprived of the context of interaction with other pathways. To investigate the dynamic interplay of signal transduction pathways, we developed ANIMO (Analysis of Networks with Interactive Modeling) [30,31,32,33]. We have previously used ANIMO to identify a new level of crosstalk between the TNFα and EGF pathways in human colon carcinoma cells [31].

Here, we tested our hypothesis that IL-1β plays a role in initiating OA by increasing WNT/β-catenin activity via iNOS by reducing DKK1 and FRZB expression in human chondrocytes. We first tested our hypothesis computationally, and validated our hypothesis experimentally in primary human chondrocytes. Using the model, we revealed a novel cross-talk between IL1β and WNT signaling in OA, which provided novel mechanistic insights in OA and for the development of novel therapeutics.

## 2. Results

### 2.1. Expression of DKK1 and FRZB Is Decreased While IL1β, NOS2/iNOS, and AXIN2 Are Increased in Human OA

**Figure 1:**
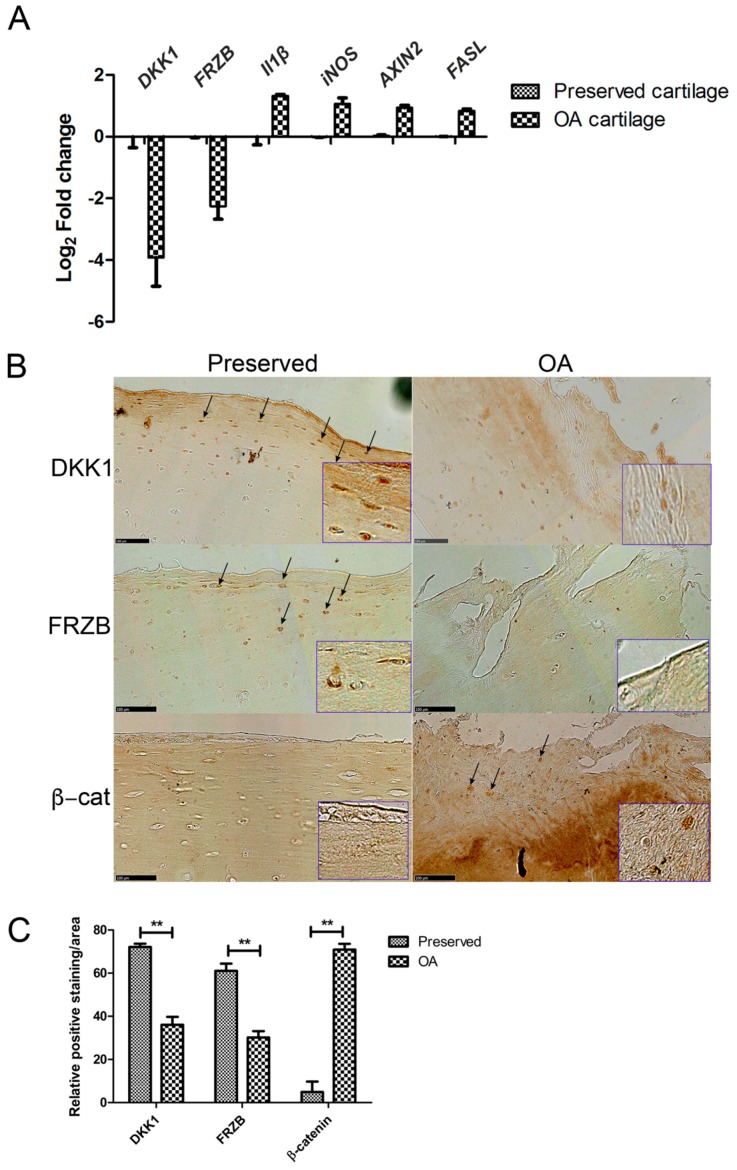
Gene and protein expression in preserved and Osteoarthritis (OA) cartilage. (**A**) RT-qPCR was performed to assess gene expression; (**B**) Immunohistochemistry (IHC) was used to visualize protein expression (arrows indicate positively stained areas). Representative pictures from one donor are shown. Images were taken using the Nanozoomer (scale bar 100 μm), magnified pictures were indicated in inserts; (**C**) Quantification of positive staining was performed by ImageJ software. ** *p* < 0.01: significant correlation.

We investigated the differences in gene and protein expression levels of IL1β, *NOS2*/iNOS, AXIN2, and FASL, and the WNT antagonists DKK1 and FRZB in human OA cartilage as compared to those that are found in macroscopically healthy looking (preserved) cartilage. In OA cartilage, DKK1 and FRZB mRNA expression was significantly decreased accompanied by overexpression of the pro-inflammatory factor *IL1B*, the gene encoding inflammatory mediator *iNOS*, the apoptotic factor FASL, and the WNT target gene *AXIN2* (axis inhibition protein 2) (Figure 1A). DKK1, FRZB, and β-catenin protein expression was detected with immunohistochemistry in paired preserved and OA cartilage specimens from ten patients. Preserved cartilage consistently demonstrated the high expression of cytosolic DKK1 and FRZB, especially in the superficial layer. In contrast, the matching OA cartilage from the same patient showed significantly decreased DKK1 and FRZB expression and increased nuclear localization of β-catenin. β-catenin was hardly detected in preserved cartilage in which high expression of DKK1 and FRZB was observed (Figure 1B, quantification of expression Figure 1C, data of each patient is shown in Figure S1). Interestingly, positive staining of DKK1 was also detected in cell clusters of some OA cartilage samples.

### 2.2. ANIMO Model Predicts That IL1β Upregulates WNT Signaling via iNOS/NO by Downregulating Expression of DKK1 and FRZB

To obtain insight into the possible mechanism by which IL1β influences WNT signaling, we generated a simplified network diagram of the WNT and IL1β signaling pathway, which was composed of key proteins. The different steps that were taken to build the model are described in the supplementary information/Figure S4. We used IL1β, IL1Receptor (IL1R), NFκB, IκB, *MMP13*, and iNOS for the IL1β pathway, and WNT, β-catenin, TCF/LEF and the antagonists DKK1 and FRZB, both as mRNA and protein, for the WNT pathway. The regulation of DKK1 and FRZB expression is summarized in a node called ‘ANAbolic Regulator’, or ANAR. The network diagram was then formalized in ANIMO, which allows us to analyze activity-based computational models. Nodes in an ANIMO network can represent proteins or mRNAs [33], while a change in node activity can describe protein phosphorylation or mRNA expression, depending on the node type. Nodes are connected by interactions (edges), which have the effect of changing the activity level of the target node if the source node is active. The speed at which an interaction occurs was abstractly modelled as either “fast” (for reactions such as phosphorylations) or “slow” (when gene transcription is involved) [30,31].

**Figure 2:**
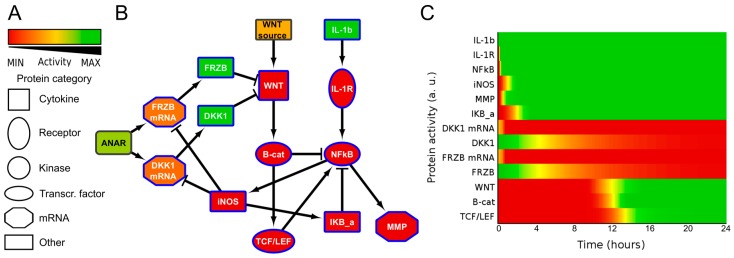
Network diagram ((**B**), model 1) and corresponding activity heatmap (**C**) of the interleukin 1β and Wingless-Type MMTV Integration Site Family (WNT) signaling pathway, in which nitric oxide synthase (iNOS) inhibits DKK1 and FRZB expression, resulting in WNT activity. (**A**) Activities are color coded from red = inactive, via yellow to green = fully active. The shape of the nodes indicate the type of protein or gene/mRNA; (**B**) Simplified network. Il-1b= IL1β, IKB-a = IκBα. For simplicity, the self-inactivating edges that formalize mRNA/protein activity life-time are not shown. The complete model, including self-inactivating edges can be found in the supplemental Figure S5A,B (interaction parameters are in Table S2, initial activities of all models are shown in Table S3).The colors of the nodes indicate their initial activity, and these colors correspond to the activity heatmap in (**C**); (**C**) activity heatmap of the network in (**B**).

It has been described for a human cancer that nitric oxide (NO) indirectly upregulates WNT/β-catenin signaling by inhibiting DKK1 [27]. We also identified that in chondrocytes IL1β treatment resulted in downregulation of both FRZB and DKK1 [25]. We therefore added a reaction from iNOS to inhibit both DKK1 mRNA and FRZB mRNA. This resulted in a small network that described additional cross-talk between ILβ and WNT signaling, model 1 (Figure 2 and Figure S5A,B). As expected, in model 1, the addition of IL1β activated WNT/β-catenin signaling via iNOS induced loss of DKK1 and FRZB.

It is often suggested that the WNT antagonists FRZB and DKK1 are functionally redundant. To visualize this, we removed the inhibition of iNOS on FRZB in our model. If DKK1 and FRZB are indeed functionally redundant, then the inhibition of only one of these factors should prevent WNT activation. Since in osteoarthritis development we found that FRZB was lost starting in grade 2, while DKK1 started to decrease in grade 1 [13], we decided to test in our model if FRZB was able to prevent WNT activity when DKK1 expression was lost. We therefore removed the inhibitory edge from iNOS to FRZB and activated only IL1β in this model, which is model 2 (Figure S5C,D). However, reduction of DKK1 expression alone did not alleviate the inhibition on WNT signaling, due to the presence of FRZB. This prediction thus suggested that IL1β would only activate WNT signaling by simultaneously downregulating both DKK1 and FRZB expression via iNOS, which we subsequently tested in the wet-lab.

### 2.3. IL1β Decreased DKK1 and FRZB Expression in a Time- but Not Dose-Dependent Manner

**Figure 3:**
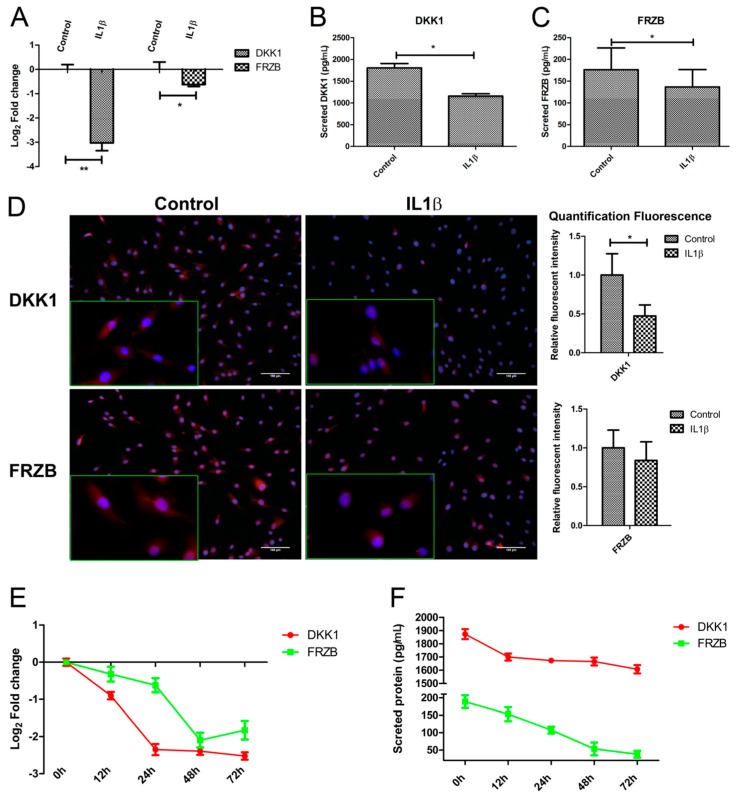
IL1β decreased expression of Dickkopf-1 (DKK1) and Frizzled related protein (FRZB) at mRNA and at the protein level. Human primary chondrocytes were treated with IL1β for 24 h. (**A**); (**B**,**C**). DKK1 and FRZB gene and protein expression were measured by qPCR and Enzyme-Linked Immunosorbent Assay (ELISA), respectively; (**D**) The expression of DKK1 and FRZB was measured by IF. DKK1 and FRZB are illustrated in red and nuclei are in blue (scale bar 100 μm), magnified pictures were indicated in inserts. Quantification of immunofluorescence intensity was performed using CellProfiler software; (**E**,**F**). IL1β decreased DKK1 and FRZB expression is time-dependent. Time-course evaluation of DKK1 and FRZB expression after IL1β stimulation. * *p* < 0.05, ** *p* < 0.01: significant correlation.

To validate that IL1β indeed regulates both mRNA and protein expression of DKK1 and FRZB in human chondrocytes (hChs), we measured the effect of IL1β on *DKK1* and *FRZB* mRNA expression by qPCR and the DKK1 and FRZB protein levels by ELISA. IL1β significantly decreased the expression of DKK1 and FRZB (Figure 3A–C).

Immunofluorescence was used to examine the localization and expression of DKK1 and FRZB in human chondrocytes. Chondrocytes in the control group demonstrated constitutive expression of DKK1 and FRZB in the cytoplasm and also in the nucleus. IL1β exposure significantly decreased DKK1 and FRZB expression, especially in the cytoplasm (Figure 3D and Figure S2A,B). IL1β exposure had a widespread effect on the expression of WNT related genes by increasing *FZD10*, *LEF1*, and *TCF4*, while downregulating the expression of the *WNT4* and the WNT inhibitor *WIF1* (Figure S2C). In addition, the effect of IL1β treatment on expression of cartilage markers, catabolic markers, and an apoptotic factor was measured by qPCR. IL1β treatment decreased *ACAN* and *COL2A1* expression, while it increased *MMP3*, *BMP2*, and FASL expression (Figure S2D).

We explored if the IL1β regulation of DKK1 and FRZB was time-dependent by performing a time-course experiment to examine the effects of IL1β treatment for up to 72 h. To ensure the efficacy of the stimulation, we measured *IL16*, *IL1B*, and *MMP3* expression, well-established target genes of IL1β. IL1β strongly induced the mRNA levels of all of these target genes, which progressively increased until at least 72 h after treatment (Figure S2E). The expression of DKK1 and FRZB in response to IL1β was time-dependent. *DKK1* and *FRZB* mRNA expression started to decrease from 12 h after stimulation and reached the lowest expression levels at 72 and 48 h, respectively (Figure 3E). The decrease in *FRZB* mRNA level occurred more slowly. In line with the qPCR results, the secreted protein levels of DKK1 and FRZB were downregulated after IL1β stimulation (Figure 3F).

Measuring the dose-dependent effects of IL1β on *DKK1* and *FRZB* mRNA expression level after 12 h using a range of 0.4 ng/mL to 50 ng/mL, revealed that IL1β treatment was already effective at the lowest concentration of 0.4 ng/mL. There was an increase of IL1β and IL6 expression with an increased concentration (Figure S2F). Exposure to IL1β at any concentration significantly downregulated DKK1 and FRZB expression both at the mRNA as well as the protein level. However, no significant difference was observed between the different IL1β concentrations (Figure S2G).

### 2.4. IL1β Decreased DKK1 and FRZB Expression through Upregulation of iNOS

**Figure 4:**
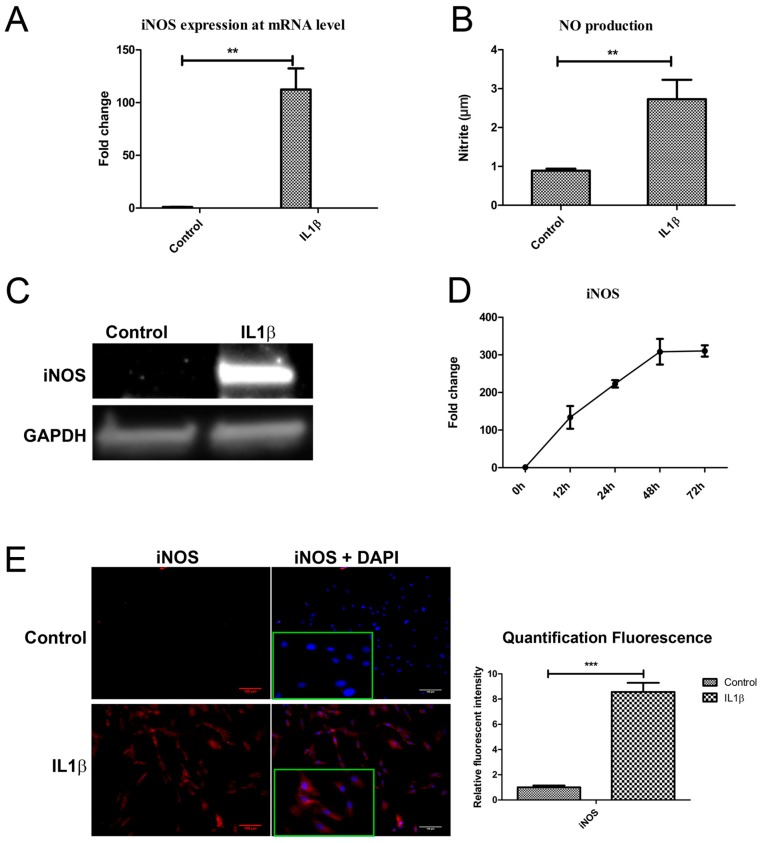
IL1β induced iNOS expression at both mRNA and protein level. (**A**,**B**) Human chondrocytes were treated with IL1β for 24 h. iNOS mRNA expression was detected by qPCR and nitric oxide (NO) production was measured by Griess assay. (**C**) Western blot was used detect iNOS protein expression. (**D**) Time course evaluation of iNOS mRNA expression after IL1β treatment. (**E**) IF was used to measure iNOS expression, magnified pictures were indicated in inserts. Scale bars 100 μm. Quantification of immunofluorescence intensity was performed by CellProfiler software. ** *p* < 0.05, *** *p* < 0.01: significant correlation.

To determine if IL1β decreased DKK1 and FRZB by upregulating iNOS, as predicted by our ANIMO model in Figure 2, we measured *NOS2*/iNOS expression and NO production 24 h after IL1β treatment. *NOS2*/*iNOS* expression at mRNA level was significantly induced by IL1β (Figure 4A). The concentration of the end product of iNOS, nitrite, was increased in cell medium, as determined by a Griess assay (Figure 4B). iNOS protein was hardly detected in relatively healthy human chondrocytes while iNOS protein was strongly increased after IL1β stimulation, as determined by western blot (Figure 4C). IL1β almost linearly (*R*^2^ = 0.9875) increased *NOS2*/iNOS expression over 48 h of stimulation (Figure 4D). Immunofluorescence staining confirmed the increase in iNOS production after exposure to IL1β for 24 h (Figure 4E). These results correspond to the data in our model, where we predicted that both DKK1 and FRZB mRNA and protein expression were affected by IL1β treatment through increase of iNOS activity.

### 2.5. Addition of 1400W Relieves the Break on DKK1 and FRZB Inhibition

**Figure 5:**
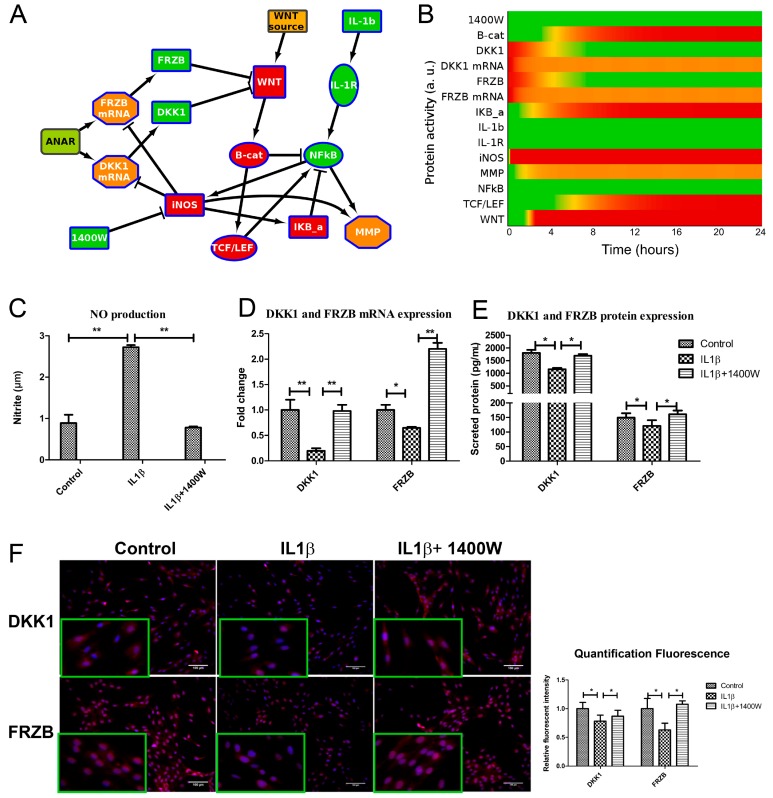
iNOS inhibitor 1400W blocked NO production and rescued the expression of DKK1 and FRZB both in the computational model and in cells. (**A**,**B**). Addition of 1400W to model 2, creating model 3, shows WNT inactivation by relieving the brake on DKK1 and FRZB expression. Activity levels at 24 h are shown. As shown in Figure 2A, Green is active, red inactive. IL-1b = IL1β, B-cat = β-catenin (protein), IKb-a = IκBα. The model with auto-inhibition edges is shown in Figure S5. (**C**) Human chondrocytes were treated with either IL1β or 1400W or both for 24 h. 1400W inhibited IL1β induced NO production, as measured by Griess assay. (**D**,**E**). The mRNA and protein expression of DKK1 and FRZB was rescued after addition of iNOS inhibitor, measured by qPCR and ELISA (**F**). The protein expression of DKK1 and FRZB was also measured by immunofluorescence (scale bar 100 μm), magnified pictures were indicated in inserts. Quantification of immunofluorescence intensity was performed by CellProfiler software. * *p* < 0.05, ** *p* < 0.01: significant correlation.

iNOS activity can be blocked using a small molecule inhibitor 1400W [34]. To in silico confirm the hypothesis that inhibiting iNOS would be sufficient to regain expression of DKK1 and FRZB, we further extended our ANIMO model. Specifically, we added the node ‘1400W’ to inhibit iNOS activity and started the model using the final activities of the model in Figure 2 (where DKK1 and FRZB expression were suppressed and the WNT pathway was active) as input settings. In this model, model 3, we observed that the addition of 1400W is sufficient to release the inhibition of DKK1 and FRZB, resulting in the inhibition of the WNT signaling pathway (Figure 5A and Figure S5E,F and Supplemental Table S2).

We experimentally tested our hypothesis that blocking iNOS using 1400W is sufficient to block WNT signaling by recovery of DKK1 and FRZB expression in human primary chondrocytes. We validated that 1400W blocked iNOS-generated nitric oxide (Figure 5C). As predicted by the model, blocking iNOS simultaneously rescued DKK1 and FRZB expression at mRNA and protein level, as was determined by qPCR (Figure 5D) and ELISA (Figure 5E), and corroborated by semi-quantified immunofluorescence microphotographs (Figure 5F and Figure S3A,B).

### 2.6. Blocking IL1β-Induced iNOS Decreased β-Catenin Expression

**Figure 6:**
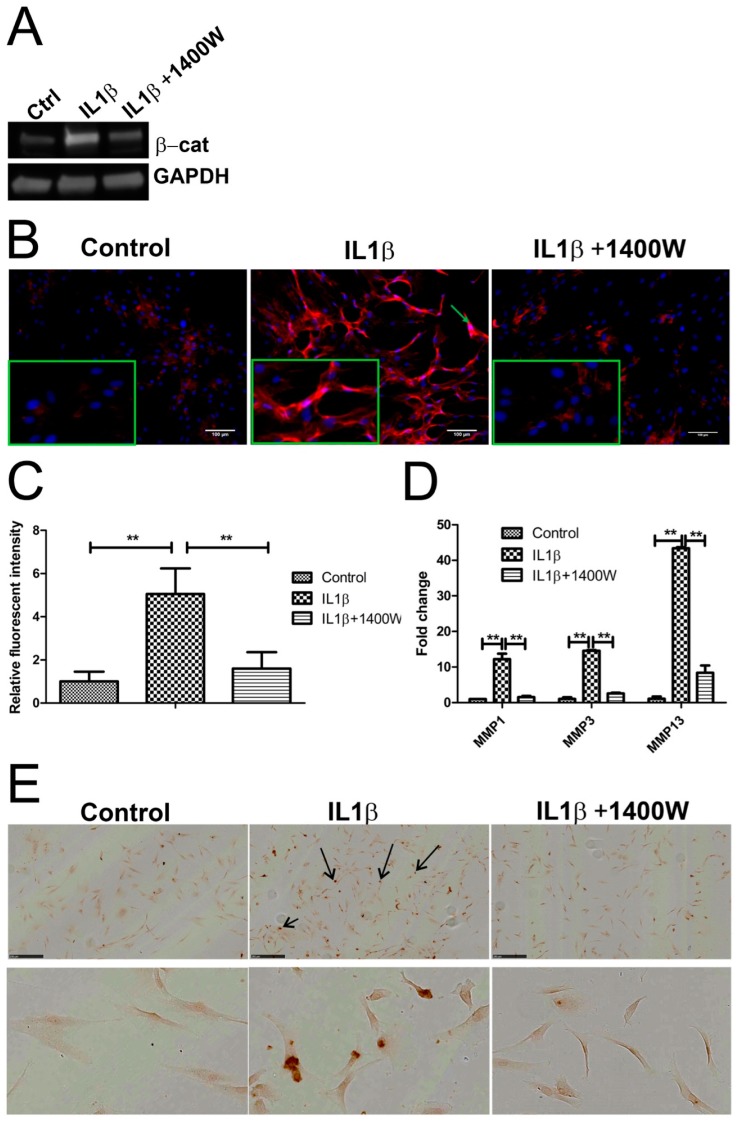
Blocking iNOS decreased β-catenin, matrix metalloproteinases (MMPs) expression, and inhibited apoptosis. (**A**,**B**) Chondrocytes were treated with 10 ng/mL recombinant human IL-1β or 100 uM iNOS inhibitor 1400W for 24 h. The protein expression of β-catenin was detected by Western blot and IF, green arrow indicated nuclear positive staining of β-catenin, magnified pictures were indicated in inserts. (**C**) Quantification of immunofluorescence intensity was performed by CellProfiler software. (**D**) MMP mRNA expression was measured by qPCR. (**E**) Apoptosis of human chondrocytes was detected using the DeadEnd colorimetric TUNEL assay. Apoptotic nuclei were stained dark brown (dark arrow). Images were taken using Hamamatsu Nanozoomer. Scale bar = 250 μm, top panel indicate overview of cell apoptosis, below panel indicates enlarged picture. ** *p* < 0.01: significant correlation.

In our models, we assumed that there was crosstalk between iNOS and the WNT signaling pathway based on the observation that IL1β treatment resulted in changes of both IL1β and WNT related genes, such as *MMP1*, *MMP3* and *MMP13*, *FRZD10*, *LEF1*, *TCF4*, *WIF1*, and *WNT4*. To determine the relationship between IL1β, iNOS, and WNT/β-catenin signaling, we measured β-catenin expression by Western blot and IF following exposure to IL1β in the presence and absence of 1400W (Figure 6A). The control group showed low level expression of membrane bound cytosolic β-catenin. IL1β highly increased cytosolic expression and nuclear localization of β-catenin and blocking IL1β-induced iNOS by 1400W decreased β-catenin expression (Figure 6B,C, and Figure S3C). In addition, we found that 1400W inhibited IL1β-induced *MMP-1*, *-3* and *-13* expression and chondrocyte apoptosis (Figure 6D,E).

### 2.7. Computational Model Highlights Role of iNOS in Regulating MMP Expression

In our computational model, *MMP* is downstream of NFκB (nuclear factor kappa-light-chain-enhancer of activated B cells) and there is no interaction between iNOS and *MMP*. Therefore, in the model, the expression of *MMP* is unaltered after 1400W addition (Figure 5B and Figure S5F). However, our wet-lab data could be explained if IL1β induced *MMP* expression is also regulated by iNOS. We therefore adapted our model by adding *MMP* downstream of iNOS and lowering the k-value for the edge from NFκB to *MMP*, resulting in the downregulation of *MMP* expression in the presence of 1400W (model 4: Figure S5G,H).

Our model explains the mechanism that iNOS is a mediator regulating both DKK1 and FRZB, as well as *MMP* expression. Combined, these are new findings in chondrocytes.

## 3. Discussion

The major and novel findings in this study are: (i) Facile computational activity-based models of signal transduction pathway crosstalk can be used to predict, visualize, and explain a cellular response; (ii) In paired samples of the same donor we showed that DKK1 and FRZB protein is highly expressed in the superficial layer of preserved cartilage, while it is lost in OA cartilage; and, (iii) IL1β induced iNOS expression in chondrocytes, which, with NO as a mediator, activated WNT signaling in primary human chondrocytes by simultaneously decreasing endogenous DKK1 and FRZB expression, while increasing *MMP* expression.

Making small activity-based computational models to describe cellular signaling pathways is an efficient and insightful way to visualize the effects of perturbation of the networks. Here, we showed that we can build relatively simple activity based networks in a facile manner and test hypotheses in silico to render visual and comprehensive results. This is particularly advantageous when discussing signaling crosstalk with people that are new to the field. While there are many more precise modeling tools (reviewed in [31]), we adopted relatively simple dynamics to mimic the interactions between only a few proteins in the networks. Using these simple dynamics, we are able to describe the timing of the interactions quite precisely when compared to our data and previous data [33].

We used ANIMO models of the IL1β/WNT signaling crosstalk to show that both DKK1 and FRZB need to be inhibited for IL1β to regulate β-catenin expression, through the re-activation of the WNT signaling pathway. In addition, because of the use of the activity-based model, we identified a link between iNOS/NO and MMP expression that would otherwise have been ignored.

It has been shown that nitric oxide influences the binding of specific transcription factors to DNA. In the case of c-MYB, c-MYB DNA binding was reduced in the presence of NO [35]. In addition, NO has been shown to reduce the binding of the transcriptional repressor Yin-Yang-1 at the Fas promotor [36]. In contrast, NO positively influenced IL8 expression in a process that was dependent on the activity of the transcription factors that normally regulated IL8 expression [37]. It is, therefore, possible that NO also influences the binding of NFκB to the promotor site of *MMP1*, *-3*, and *-13*, so that *MMP1*, *-3* and *-13* expression is higher in the presence of NO, which is what we observe. Inhibiting the NO production by 1400W would then result in a lower binding efficiency of NFκB binding to its target genes, thereby decreasing the expression of *MMP1*, *-3*, and *-13*.

In this manuscript, we chose to make the model as small as possible was based on the principle of Occam’s razor: we built a model that involves a minimal amount of players and can still describe the effects that we observed thereafter in experimental data. One has to keep in mind that some edges in the model, such as the one connecting WNT and β-Catenin, do not necessarily represent actual interactions. What those edges represent is the “net effect” of the activation of some significant players. In the example, the edge represents the fact that β-Catenin concentration increases when WNT is available. We avoided the inclusion of all the intermediate steps in that process, as their presence would have increased the number of nodes in the network without increasing the capability of the model to explain our hypothesis. However, for other applications, for example when precise information on protein interactions is investigated, more detailed modeling may be necessary ([38], and reviewed in [31]).

In previous work, we found that simultaneous inhibition of endogenous DKK1 and FRZB using neutralizing VHH llama antibodies, inhibited redifferentiation of human chondrocytes and induced hypertrophic differentiation in co-cultures of human mesenchymal stem cells and human chondrocytes, indicating that the simultaneous expression of DKK1 and FRZB is important for chondrocyte development and the prevention of hypertrophic differentiation [39]. Both DKK1 and FRZB are antagonists of WNT signaling, but they antagonize WNT signaling through different mechanisms. DKK1 inhibits the canonical pathway [40,41] and FRZB antagonizes WNT signaling of both canonical and noncanonical pathways [42,43,44,45]. The canonical pathway regulates proliferation and the noncanonical pathway regulates differentiation [46]. In OA cartilage, chondrocytes not only show abnormal proliferation but also hypertrophic differentiation and dedifferentiation, suggesting that both canonical and noncanonical WNT pathways can be activated due to the inhibition of DKK1 and FRZB expression.

We find that the expression of *DKK1* and *FRZB* decreased while *IL1B*, *NOS2*/iNOS, and *AXIN2* was increased in OA cartilage. In addition, we found that DKK1 and FRZB protein is highly present in relatively healthy cartilage but is lost in the paired OA cartilage. Both of these findings are in line with previous work of our group and others [12,16,47]. In contrast, β-catenin showed high expression in OA and low expression in the paired preserved samples. This indicates that diminished DKK1 and FRZB expression favors the activation of canonical WNT signaling and consequently contributes to OA. Interestingly, in some patients, DKK1 positive staining was also observed in some of the cell clusters in the middle layer of OA cartilage. It has been reported that the overexpression of cartilage markers, such as *SOX9*, *ACAN*, and COLII is observed in cell clusters in OA [48]. Given the anabolic role of DKK1 in cartilage homeostasis, it is not surprising that some repopulated cells produce DKK1 to antagonize WNT signaling in OA cartilage.

IL1β is known as a non-specific activator of WNT signaling [49,50]. We previously showed that the expression of *DKK1* and *FRZB* mRNA decreased after exposure to IL1β, and that IL1β induces β-catenin accumulation, which may be through inhibition of WNT inhibitors in human chondrocytes [25]. We showed here that in human chondrocytes, IL1β significantly downregulated DKK1 and FRZB, and that this regulation is time dependent, but not dose dependent. In addition, IL1β induced expression of several WNT related gene such as *FRZD10*, *LEF1*, and *TCF4*, while expression of the WNT inhibitor *WIF1* was reduced. This matches with findings in a cancer cell line [27]. It has been shown that inflammatory factors reduce cartilage proteoglycan synthesis [22] and induce chondrocyte hypertrophy [51,52,53,54]. Indeed, we show that IL1β inhibited the expression of the cartilage markers *ACAN* and *COL2A1*, while it induced the expression of the hypertrophic markers *COL10A1*, *MMP13*, and *BMP2*. It thus appears that IL1β induces chondrocytes to switch their stable articular phenotype to a hypertrophic state by decreasing DKK1 and FRZB expression via iNOS.

In cancer cells, IL1β induced nitric oxide production and that this upregulated WNT/β-catenin signaling by inhibiting DKK1 expression [27]. We found that blocking iNOS rescued the expression of both of DKK1 and FRZB, and also inhibited IL1β-induced MMP expression and chondrocyte apoptosis. This matches with work by Pelletier et al., in which another iNOS inhibitor, L-NIL, was shown to reduce *MMP1* and *MMP3* expression [55] and inhibits chondrocyte apoptosis in OA dogs [56]. It is of note that other mechanisms might contribute to the inhibition of DKK1 and FRZB. For example, our group has previously shown that abnormal mechanical loading and tonicity decreased DKK1 and FRZB expression in human chondrocytes [12].

## 4. Materials and Methods

### 4.1. ANIMO

ANIMO is a software tool that is designed to be used in biological research and operates as an application in Cytoscape [57]. The supplementary methods describe the use of ANIMO. ANIMO networks can include activations (→) and inhibitions (─┤), which will increase (resp. decrease) the activity level of the target node if the source node is active. For example, A → B will increase the activity level of B if A is active. The speed at which an interaction occurs is defined by its *k* parameter, which can be estimated qualitatively by choosing among a pre-defined set of options (very slow, slow, medium, fast, very fast), or by directly inputting a numerical value. We initially used only the qualitative interaction speeds ‘very slow, slow, medium, fast, very fast’, and successively adapted some k-values to obtain more realistic behaviour from the network. In particular, we lowered the speed of some self-inhibitions to values below the ‘very slow’ speed. We use these interactions to represent the processes of degradation/deactivation of proteins that constantly happen in the cell, but at a rate that is normally (much) lower than the production/activation of their targets.

In all of the models, the colors of the nodes represent its activity level at a certain time-point, and correspond to the colors/activities at that time point in the heat-map. In most images, the activities at the beginning of simulation is shown, unless otherwise stated. The colors thus represent the starting activities of the nodes in the network. Instructions on how to install and use ANIMO can be found on our web site [58]. Also, all of the published models are freely available on this site.

### 4.2. Human Cartilage

The collection and use of human cartilage was approved by a medical ethical committee (METC) of Zorggroep Twente, The Netherlands. Cartilage was obtained from 10 patients with OA undergoing total knee replacement surgery (seven female, 1one male, two unknown, median age 62 ± 12). Preserved cartilage samples were isolated from macroscopically intact areas and OA cartilage specimens were isolated from areas that were affected by OA, as described in supplemental methods and in [13].

Cartilage samples were collected into 10 mL tubes and were washed twice with PBS. For RNA isolation, subchondral bone was removed from the cartilage, and samples were cut into small pieces (1–2 mm) and quickly snap frozen into liquid nitrogen. Samples were stored at −80 °C. For histology, cartilage samples were fixed using 10% phosphate buffered formalin (pH = 7, Sigma Aldrich, St. Louis, MO, USA) overnight at 4 °C, decalcified for four weeks in 12.5% (*w*/*v*) EDTA solution containing 0.5% phosphate buffered formalin (pH 8.0), dehydrated using graded ethanol, and embedded in paraffin.

### 4.3. RNA Isolation and qPCR Analysis

Total RNA was isolated using TRIzol (ThermoFisher Scientific, Waltham, MA, USA, for details see supplementary methods and [13]). mRNA was isolated from cells using the NucleoSpin RNA II kit (Macherey-Nagel, Dueren, Germany), according to the manufacturers protocol. For cartilage samples: Cartilage pieces were transferred into a pre-cooled Cryo-Cup Grinder for crushing. The obtained cartilage powder was collected into 50 mL tubes and samples were weighed. One mL TRIzol reagent per 50–100 mg sample was added.

The isolated RNA was treated with RNase-free DNase I (Invitrogen Life Technologies, ThermoFisher Scientific, MA, USA). cDNA was obtained from 1 μg of RNA with a cDNA synthesis kit (BIO-RAD, Hercules, CA, USA). QPCR was performed using SYBR Green sensimix (Bioline, London, UK) in the Bio-Rad CFX96 (Bio-Rad, Hercules, CA, USA). For each reaction, a melting curve was generated to test for primer dimer formation and non-specific priming. *GAPDH* was used for gene expression normalization. Mean fold change of gene expression was transformed to log_2_, which was plotted. Primer sequences are listed in Supplemental Table S1.

### 4.4. Immunohistochemistry (IHC) and Immunofluorescence

Immunohistochemical staining of DKK1, FRZB, and β-catenin was performed on 5 μm tissue sections, as previously described [13]. Rabbit polyclonal DKK1 (sc-25516), rabbit polyclonal FRZB (from sc-13941), rabbit polyclonal iNOS (sc-651), all from Santa Cruz Biotechnology, Dallas, TX, USA, and rabbit polyclonal β-catenin (LS-C203657, LifeSpan Biosciences, Seattle, WA, USA) were diluted 1:500 in 5% BSA in PBS and incubated overnight at 4 °C. Non-immune controls were performed without primary antibody. A biotinylated secondary antibody was diluted 1:500 and HRP-Streptavidin was added. For visualization, DAB substrate kit was used (ab64238, Abcam, Cambridge, UK). Quantification was performed using ImageJ software (FIJI) [59].

For immunofluorescence, hChs were seeded at a density of 10^4^ cells/cm^2^ on coverslips and cultured for 24 h in the presence or absence of 10 ng/mL of IL1β. Samples were washed three times with PBS, fixed with 10% formalin for 30 min, and permeablized with 0.5% triton X-100 in PBS for 15 min at RT. Samples were blocked in 1.5% of BSA in PBST for 1 h, then incubated with specific primary antibody against DKK1, FRZB, iNOS, or β-catenin (Cat.# and supplier, see above) overnight at 4 °C. Cells were rinsed with PBS for three times, 5 min/time. Then, Alexa^®^ Fluor 546-labelled goat anti-rabbit or anti-mouse antibody in 1.5% BSA in PBST was added for 2 h at RT. Cells were rinsed with PBS and mounted in mounting medium with DAPI. Slides were imaged using a BD pathway confocal microscope.

### 4.5. Human Primary Chondrocyte Isolation and Cell Culture

Human primary articular chondrocytes (hChs) were isolated from macroscopically healthy looking areas of OA cartilage from patients undergoing total knee replacement and were cultured in DMEM (Gibco) supplemented with 10% Fetal Bovine Serum (FBS), 100 U/mL Penicillin, 100 mg/mL Streptomycin, 0.4 mM proline, 0.2 mM ascorbic acid diphosphate, and 1% nonessential amino acids. Passage two cells were used for all experiments.

### 4.6. Recombinant Proteins and Reagents

Recombinant human IL-1β was obtained from R&D Systems. The inhibitor of nitric oxide, 1400W, was purchased from Cayman Chemical (Ann Arbor, MI, USA).

### 4.7. Enzyme-Linked Immunosorbent Assay (ELISA)

Cell culture medium was collected. Secreted DKK1 and FRZB protein concentrations were determined by ELISA following the manufacturer’s instructions (Cat.#: DY1906 for DKK1; DY192 for FRZB, R&D systems).

### 4.8. Western Blotting

Total cell proteins were collected in RIPA buffer (Cell Signaling Technology, Danvers, MA) supplemented with Halt protease and phosphatase inhibitor cocktail (ThermoFisher Scientific, MA, USA). The specific antibodies used for Western blot analysis including: Anti-iNOS (sc-651, Santa Cruz Biotechnology, TX, USA), anti-GAPDH (G8795, Sigma-Aldrich, St. Louis, MO, USA), and anti-β-catenin (LS-C203657, LifeSpan Biosciences, Seattle, WA, USA).

### 4.9. NO Production Assay

Cell supernatant was collected and quantified for nitrite using the Griess reaction as previously described [60].

### 4.10. Immunofluorescent Staining

hChs were seeded at a density of 10^4^ cells/cm^2^ on coverslips and cultured for 24 h in the presence or absence of 10 ng/mL of IL1β. Samples were fixed with 10% formalin. For the detection of DKK1, FRZB, iNOS, or β-catenin, specific antibodies were used (Cat.# and supplier, see above) As a secondary antibody, Alexa^®^ Fluor 546-labelled goat anti-rabbit or anti-mouse antibody was added. Cells were mounted in mounting medium with DAPI and imaged using a BD pathway confocal microscope.

Quantification was performed using CellProfiler 2.2.0 software. The fluorescence intensity of each cell was measured and the mean intensity of all cells was calculated as pixels/cell. The intensity of fluorescence of each experimental group was normalized to the control group. Graphs represent relative fluorescent intensities.

### 4.11. Apoptosis Assay

Human chondrocytes were exposed to 10 ng/mL of recombinant human IL-1β and/or 100 uM of iNOS inhibitor 1400W for 48 h. Apoptosis of human chondrocytes was detected using the DeadEnd colorimetric TUNEL assay (Promega, Madison, WI, USA) following the manufacturer’s procedure. Apoptotic nuclei were stained dark brown.

### 4.12. Statistical Analysis

Three donors were used as biological triplicates, with at least three technical replicates per experiment. Statistical analysis between groups was performed using student’s *t*-test. The difference between multiple groups was tested using one-way ANOVA and Tukey post hoc analysis. *p* < 0.05 was considered statistically significant.

## 5. Conclusions

Our data suggest a pivotal role of iNOS/NO in the inflammatory response of human OA through indirect upregulation of WNT signaling. Blocking NO production immediately after trauma using pharmacological methods may inhibit the loss of the articular phenotype in OA by preventing downregulation of the expression of DKK1 and FRZB.

## Supplementary Materials

Supplemental information can be found at www.mdpi.com/1422-0067/18/11/2491/s1.

## Abbreviations

ACAN	Aggrecan
ANAR	ANAbolic regulator
ANIMO	Analysis of networks with interactive modeling
*AXIN2*	Axis inhibition protein 2, mRNA
*COL2A1*	Collagen 2 variant a1
DKK1	Dickkopf-1
EGF	Epidermal growth factor
*FASL*	Fas ligand/CD95L-mRNA
FRZB	Frizzled related protein
*FZD10*	Frizzled-10-mRNA
hCh	Human chondrocyte
IHC	Immunohistochemistry
IL	Interleukin
IL1R	IL1Receptor
iNOS/*NOS2*	nitric oxide synthase
IκB	Inhibitor of κB
LEF	Lymphoid enhancer-binding factor
*MMP3*	Matrix metalloproteinase 3- mRNA
NF-κB	nuclear factor kappa-light-chain-enhancer of activated B cells/nuclear factor kappa B
NO	Nitric oxide
OA	Osteoarthritis
SNP	Single nucleotide polymorphisms
TCF4	Transcription factor 4
TNFa	Tumor necrosis factor α
*WIF1*	Wnt inhibitory factor 1-mRNA
WNT	Wingless-type MMTV integration site family
