# Supplementary material for: Nitric Oxide Mediates Crosstalk between Interleukin 1β and WNT Signaling in Primary Human Chondrocytes by Reducing DKK1 and FRZB Expression
Source: Int J Mol Sci. 2017 Nov 22;18(11):2491. doi: 10.3390/ijms18112491 (PMC5713457; doi:10.3390/ijms18112491)
Supplement: Supplementary file 1 [file ijms-18-02491-s001.pdf]

Supplemental figure S1. Immunohistochemistry of all cartilage donors used in this study.

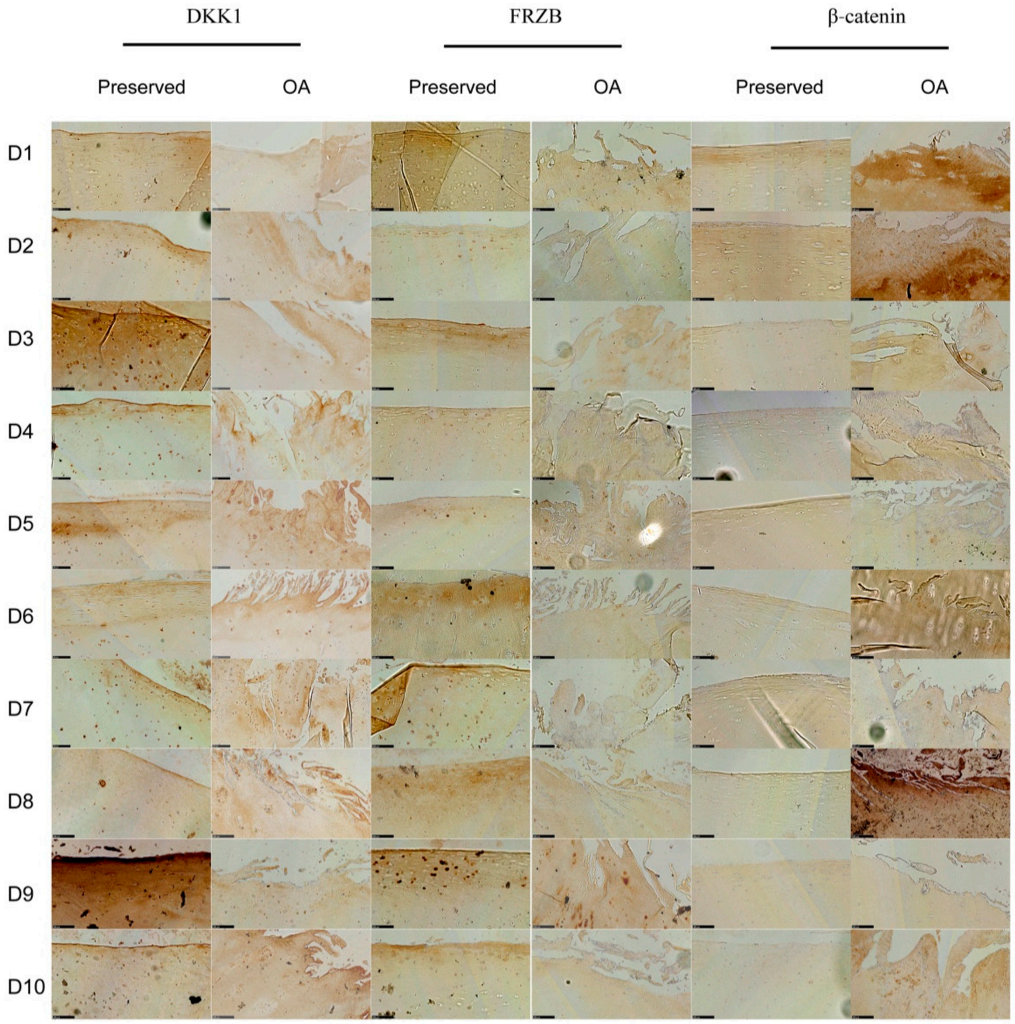

Supplemental figure S1. Protein expression of DKK1, FRZB and β-catenin was detected by IHC in all donors. Images were taken using the Nanozoomer (scale bar 100μm). D1-D10=Donor 1-10.

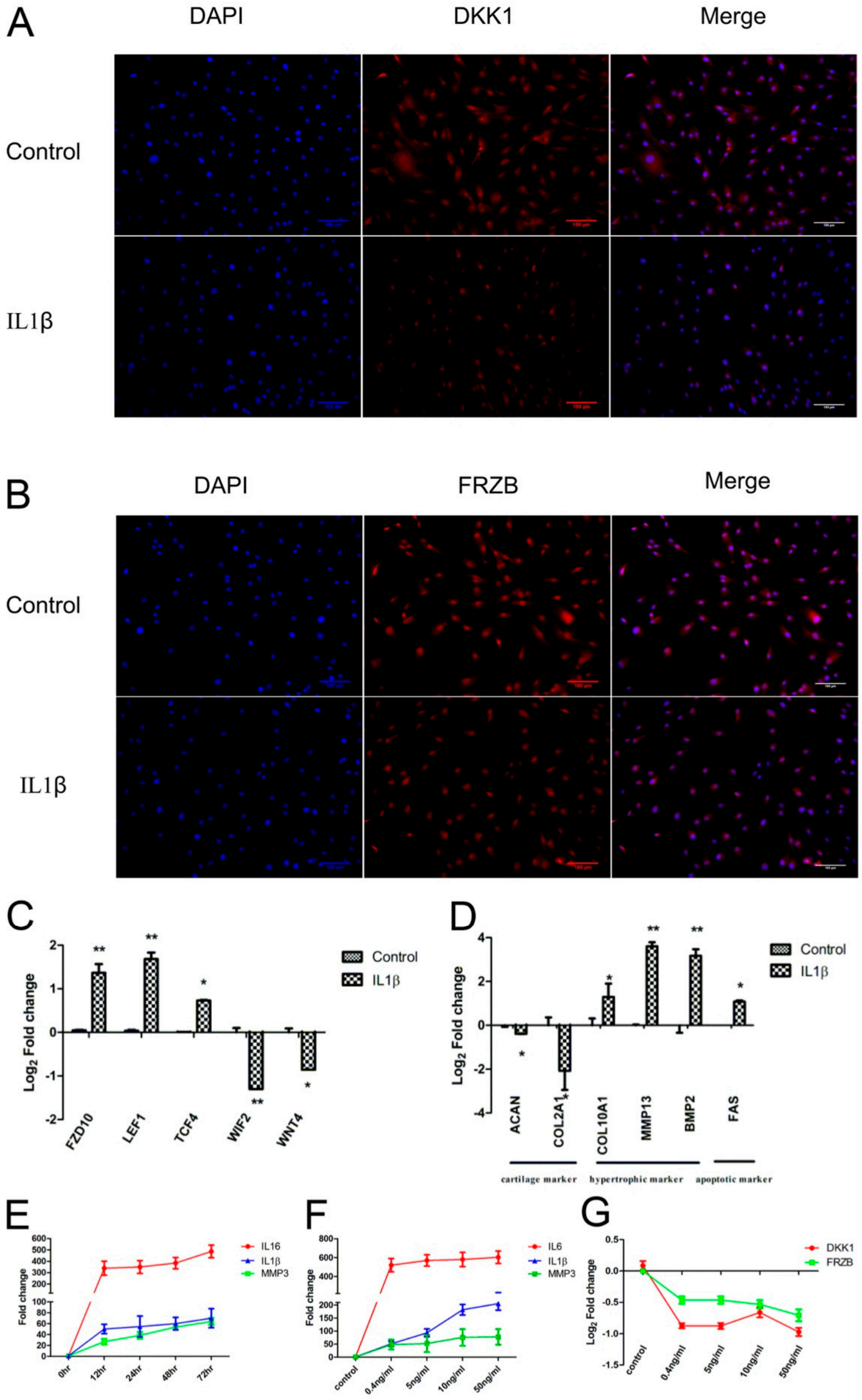
A
DAPI
DKK1
Merge
Control
IL1β
B
DAPI
FRZB
Merge
Control
IL1β
C
Log2 Fold change
FZD10
LEF1
TCF4
WIF2
WNT4
Control
IL1β
D
Log2 Fold change
ACAN
COL2A1
COL10A1
MMP13
BMP2
FAS
cartilage marker
hypertrophic marker
apoptotic marker
Control
IL1β
E
Fold change
IL16
IL1β
MMP3
0hr
12hr
24hr
48hr
72hr
F
Fold change
IL6
IL1β
MMP3
control
0.4ng/ml
5ng/ml
10ng/ml
50ng/ml
G
Log2 Fold change
DKK1
FRZB
control
0.4ng/ml
5ng/ml
10ng/ml
50ng/ml

Supplemental figure S2. The effects of IL1β on DKK1 and FRZB and Cartilage and WNT related genes. A. DKK1 and B. FRZB are illustrated in red and nuclei are in blue (DAPI). C, D. IL1β decreased mRNA expression of cartilage markers *ACAN* and *COL2A1* while increased hypertrophic and apoptotic markers, WNT receptor *FRZD10* and transcription factors *TCF4* and *LEF1* were induced by IL1β. WNT inhibitor *WIF2*, *WNT4* expression was decreased upon IL1β stimulation. E,F. qPCR was used to measure the expression of IL1β target gene *IL16*, *IL1β* and *MMP3*; G. Expression of WNT antagonists *DKK1* and *FRZB* mRNA at indicated time and dose point after IL1β treatment.

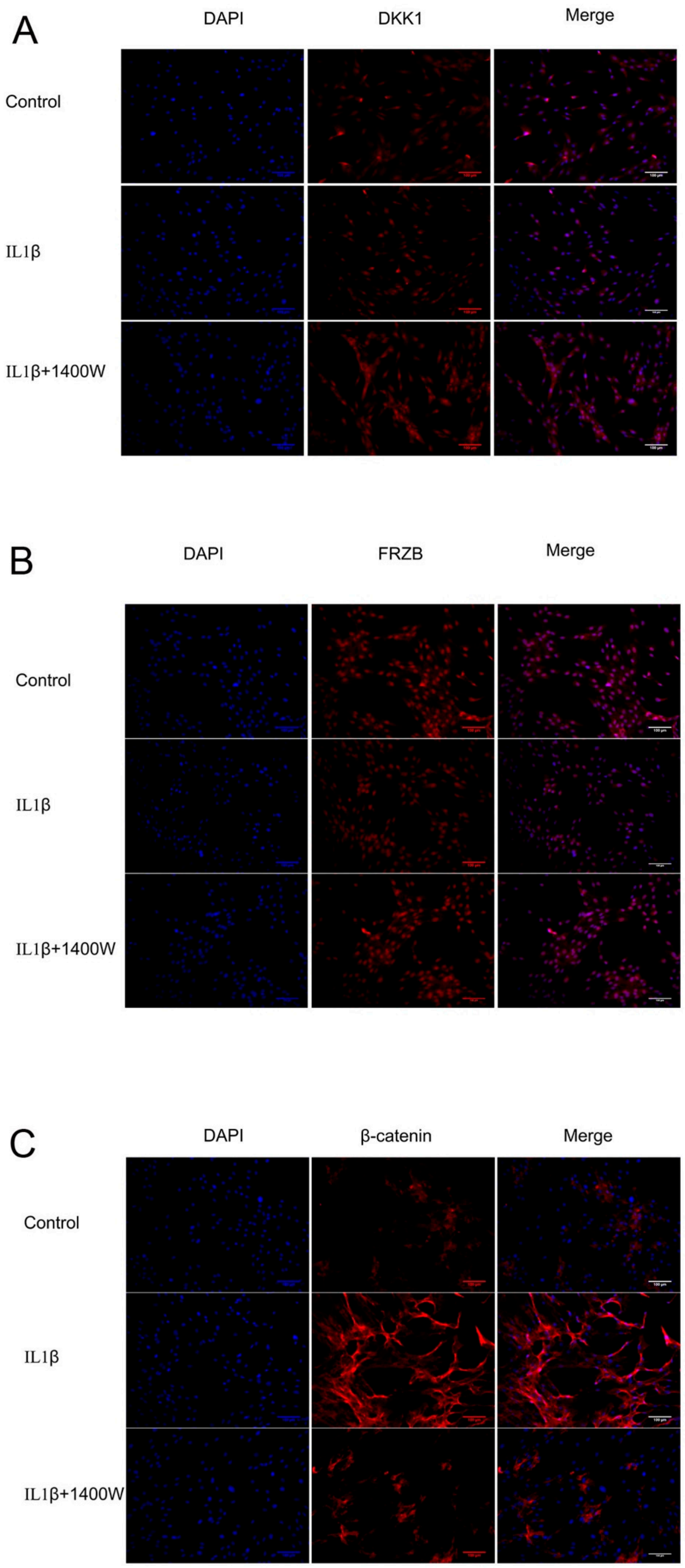
A
DAPI
DKK1
Merge
Control
IL1β
IL1β+1400W
B
DAPI
FRZB
Merge
Control
IL1β
IL1β+1400W
C
DAPI
β-catenin
Merge
Control
IL1β
IL1β+1400W

Supplemental figure S3. The effects of IL1β and iNOS inhibitor on DKK1 and FRZB and β-catenin expression visualized by immunofluorescence. A. DKK1 and B. FRZB and C. β-catenin in red and nuclei are in blue.

Supplemental table S1. Primer sequences. PCR Reactions were carried out using the Bio-Rad CFX96 (Bio-Rad, Hercules, CA) under the following conditions: cDNA was denatured for 5 min at 95°C, followed by 39 cycles consisting of 15s at 95°C, 15s at 60°C and 30s at 72°C. For each reaction, a melting curve was generated to test primer dimer formation and non-specific priming. Gene expression was normalized using GAPDH as housekeeping gene.

Gene Name	Primer Sequence	Product Size	Annealing Temperature
GAPDH	Forward primer: 5′ CGCTCTCTGCTCCTCCTGTT 3′	81	60
	Reverse primer: 5′ CCATGGTGTCTGAGCGATGT 3′		
IL6	Forward primer: 5'GGCACTGGCAGAAAACAACC 3'	85	60
	Reverse primer: 5'GCAAGTCTCCTCATTGAATCC 3'		
TCF4	Forward primer: 5′ GCACTGCCGACTACAATAGG 3′	98	60
	Reverse primer: 5′ CTGCATAGCCAGGCTGATTC 3′		
MMP1	Forward primer: 5′GGGAGATCATCGGGACAACTC 3′	72	60
	Reverse primer: 5′ GGGCCTGGTTGAAAAGCAT3′		
MMP3	Forward primer: 5′TGGCATTCAGTCCCTCTATGG 3′	116	60
	Reverse primer: 5′ AGGACAAAGCAGGATCACAGTT3′		
MMP13	Forward primer: 5′AAGGAGCATGGCGACTTCT 3′	72	60
	Reverse primer: 5′ TGGCCCAGGAGGAAAAGC3′		
IL1β	Forward primer: 5′ TCCCCAGCCCTTTTGTTGA3′	91	60
	Reverse primer: 5′ TTAGAACCAAATGTGGCCGTG3′		
INOS	Forward primer: 5′ CTCATCTCCCGTCAGTTGGT 3′	168	60
	Reverse primer: 5′ AGGGACAAGCCTACCCCTC 3′		
DKK1	Forward primer: 5′ AGTACTGCGCTAGTCCCACC 3′	172	60
	Reverse primer:5′ TCCTCAATTTCTCCTCGGAA 3′		
FRZB	Forward primer: 5′ACGGGACACTGTCAACCTCT 3′	155	60
	Reverse primer: 5′ CGAGTCGATCCTTCCACTTC 3′		
FASL	Forward primer: 5′CTCTTGAGCAGTCAGCAACAGG 3′	107	60
	Reverse primer: 5′ ATGGCAGCTGGTGAGTCAGG3′		
AIXN2	Forward primer: 5′ AGTGTGAGGTCCACGGAAAC 3′	103	60
	Reverse primer: 5′ CTGGTGCAAAGACATAGCCA 3′		
BMP2	Forward primer: 5′ GCTAGACCTGTATCGCAGGC 3′	74	60
	Reverse primer: 5′ TTTTCCCACTCGTTTCTGGT 3′		
FZD10	Forward primer: 5′ AAAGTGTCTCTGCCAACCTA3′	205	60
	Reverse primer: 5′ AGAAACCCTTCAGTGCTACA3′		
LEF1	Forward primer: 5' CGAAGAGGAAGGCGATTTAG 3'	109	60
	Reverse primer: 5' CTGAGAGGTTTGTGCTTGTC 3'		
WIF1	Forward primer 5' TCAGAAAAGCGCAACAGAGA 3'	132	60
	Reverse primer: 5' TGATGCCTTTATCCAGGGAG 3'		
WNT4	Forward primer: 5′ CTCGTCTTCGCCGTCTTCT3′	101	60
	Reverse primer: 5′ AGTTTCTCGCACGTCTCCTC3′		
ACAN	Forward primer: 5′ TTCCCATCGTGCCTTTCCA 3′	121	60

	Reverse primer: 5′ AACCAACGATTGCACTGCTCTT 3′		
COL2A1	Forward primer: 5′ GGCGGGGAGAAGACGCAGAG 3′	129	60
	Reverse primer: 5′ CGCAGCGAAACGGCAGGA 3′		
FAS	Forward primer: 5'CAACAACCATGCTGGGCATC3'	99	60
	Reverse primer:5'TGATGTCAGTCACTTGGGCATTAAC3'		
COL10A1	Forward primer: 5′ GAACTCCCAGCACGCAGAAT 3′	121	60
	Reverse primer: 5′ CCTGTGGGCATTTGGTATCG 3′		

Building an ANIMO model for investigating signaling cross-talk

Nodes in an ANIMO network can represent both proteins directly involved in signal transduction (e.g. kinases) and other related entities, such as cytokines, genes and mRNA. An *activity level* is associated to each node, to represent for example the relative amount of phosphorylated kinase or the concentration of mRNA. The activity level of a node can be altered by *interactions* with other nodes. ANIMO networks can include activations (→) and inhibitions (– ┤), which will increase (resp. decrease) the activity level of the target node if the source node is active. For example, A → B will increase the activity level of B if A is active. The speed at which an interaction occurs is defined by its *k* parameter, which can be estimated qualitatively by choosing among a pre-defined set of options (*very slow*, *slow*, *medium*, *fast*, *very fast*) or by directly inputting a numerical value. Using the indicated qualitative choices already leads to useful models: e.g. a *slow* interaction to represent the production of a protein, and a *fast* one for a post-translational modification such as phosphorylation is sufficient to provide a realistic behavior in a network with the proper node topology [30-32].

Step 1: Building the IL1 pathway

When building our model in ANIMO we aimed to make it as simple as possible, using the minimal amount of proteins and interactions necessary to describe a process. The canonical IL1β/ NFκB pathway is important for inflammation. We therefore drew nodes to represent IL1β, IL1R, NFκB and its inhibitor IKβa, and iNOS, see Figure S4B,C. In our models, we assume that there are 2 types of reactions: fast reactions for post-translational modifications, such as phosphorylation, and slow reactions where gene transcription occurs. We therefore added reactions between nodes using these 2 types of reaction speed with a "scenario 1" setting. We also add auto-inhibition to indicate inhibition as described in the literature for e.g. receptor internalization, phosphatase activity and, in the case of NFκB, nuclear export as regulated by IκB.

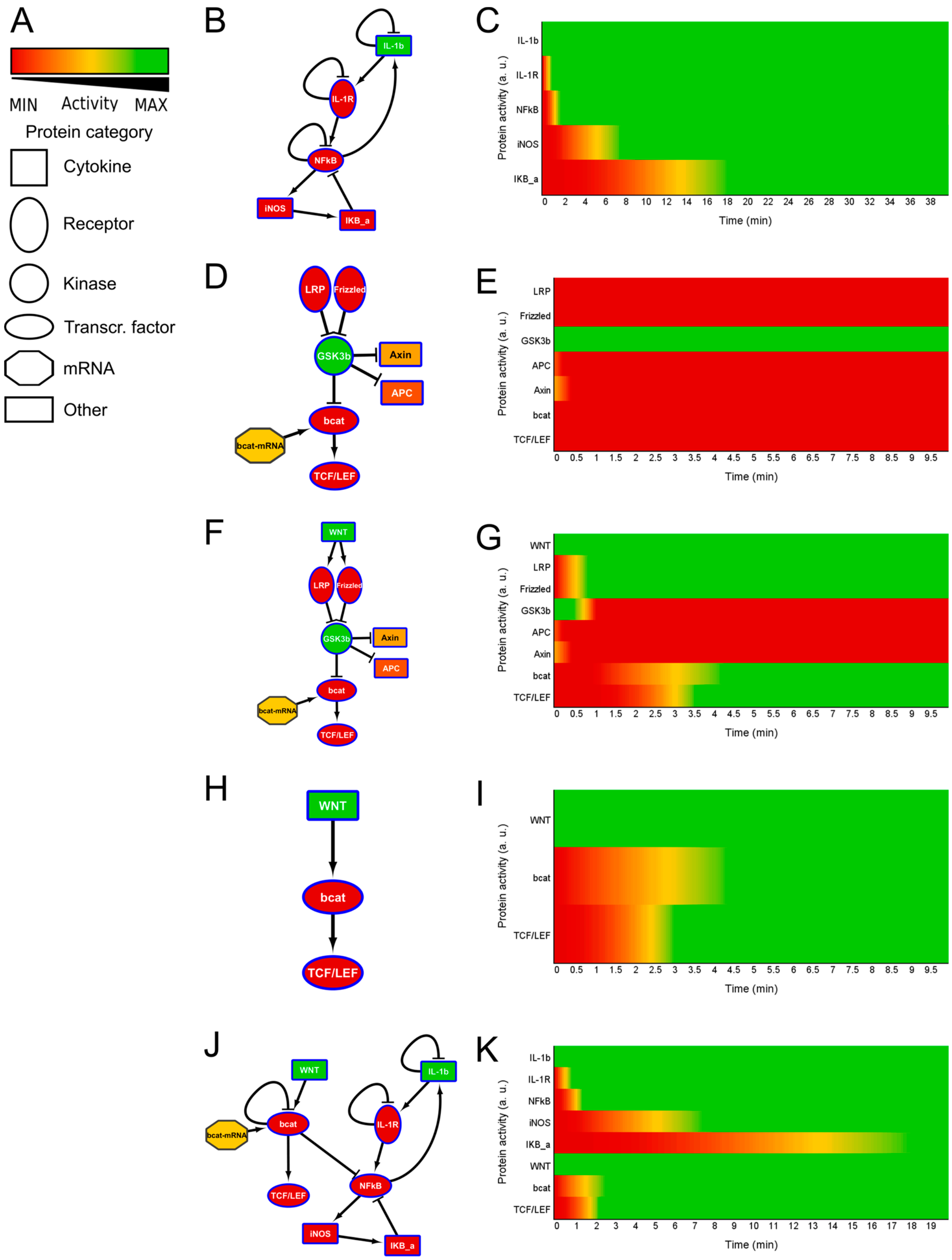

Supplemental figure S4. IL1 and WNT signaling in ANIMO models. A. Legend. Shape of the nodes defines the type of node, activity is on a scale of red = inactive, to green = active. B. IL1β activates iNOS via NFκB after addition of IL1β. Green is active and red inactive. C. the heatmap indicates protein activity in time (relative time units); D, E. Model of an inactive WNT pathway where the destruction complex, consisting of GSK3β, AXIN2 and APC, is active resulting in degradation of β-catenin. F, G. When WNT is added to the network GSK3β is inactivated thereby alleviating the downregulation of β catenin. β-catenin then accumulates and can bind to TCF/LEF, activating this transcriptional complex. H, I. Simplifying the WNT signaling pathway results in WNT activating β-

catenin, and thereby TCF/LEF, at similar rates; J, K. Simple diagram of the active IL1 and WNT pathways.

Step 2: Modeling the WNT pathway

In this model, we only consider WNT signaling via β-catenin, since DKK1 is a WNT antagonist that functions by binding to LRP5/6, which are co-factors for the WNT receptors FRIZZLED, that activate β-catenin by inhibition of the β-catenin destruction complex.

In order to represent the canonical WNT/β-catenin signaling pathway we have to consider that when no WNT ligand is present the destruction complex is active. Its function is to destroy the ubiquitously expressed β-catenin (Figure S4D,E). As an effect, β-catenin is inactive.

However, when WNT is present, GSK3β is inactivated, AXIN and APC are recruited to the receptor complex, indicated by the inhibiting edges (Figure S4F,G). As an effect, β-catenin is not degraded and accumulates in the cytoplasm and translocates to the nucleus to form a transcriptional complex with the TCF/LEF transcription factors.

Although we can model this quite well, it would be easier to simplify the exact protein interactions so that in absence of WNT, β-catenin is inactive and in presence of WNT β-catenin is active. We therefore chose to model the WNT signaling pathway as shown in Figure S4H, I. As can be seen in the activity plots on the right, the timing and intensity of β-catenin and TCF/LEF is similar to Figure S4G.

Step 3: Combining the IL and the WNT pathways

We added the WNT network representation as in Figure S4H to the IL1β model in Figure S4B, creating the model in Figure S4J. As can be seen from the network diagram, the only interaction between these networks is that β-catenin downregulates NFκB. It is known that the WNT signaling pathway influences the IL1β pathway by β –catenin inhibiting NFκB [26]. We therefore added an edge from β-catenin to NFκB (Figure S4J). This addition slows down, but does not completely inhibit NFκB activation.

Step 4: Adding the WNT antagonists DKK1 and FRZB

To test our hypothesis that IL1β activates WNT signaling through DKK1 and FRZB repression (see main manuscript), we added nodes to the network for 'DKK1 mRNA' and 'FRZB mRNA'. The upstream signals activating the transcription of these genes is not completely clear. However, we do know that in healthy articular cartilage these factors are expressed, whereas in OA the expression of these genes is greatly reduced [12,13]. We therefore added a node "anabolic regulator' or 'ANAR' to our model that induces the transcription of *DKK1* and *FRZB.* We then added the nodes DKK1 and FRZB protein, that both function to antagonize WNT signaling. If there is no cross-talk from the IL1β pathway to the WNT pathway, WNT and β-catenin become inactive due to the presence of DKK1 and FRZB (Figure S5).

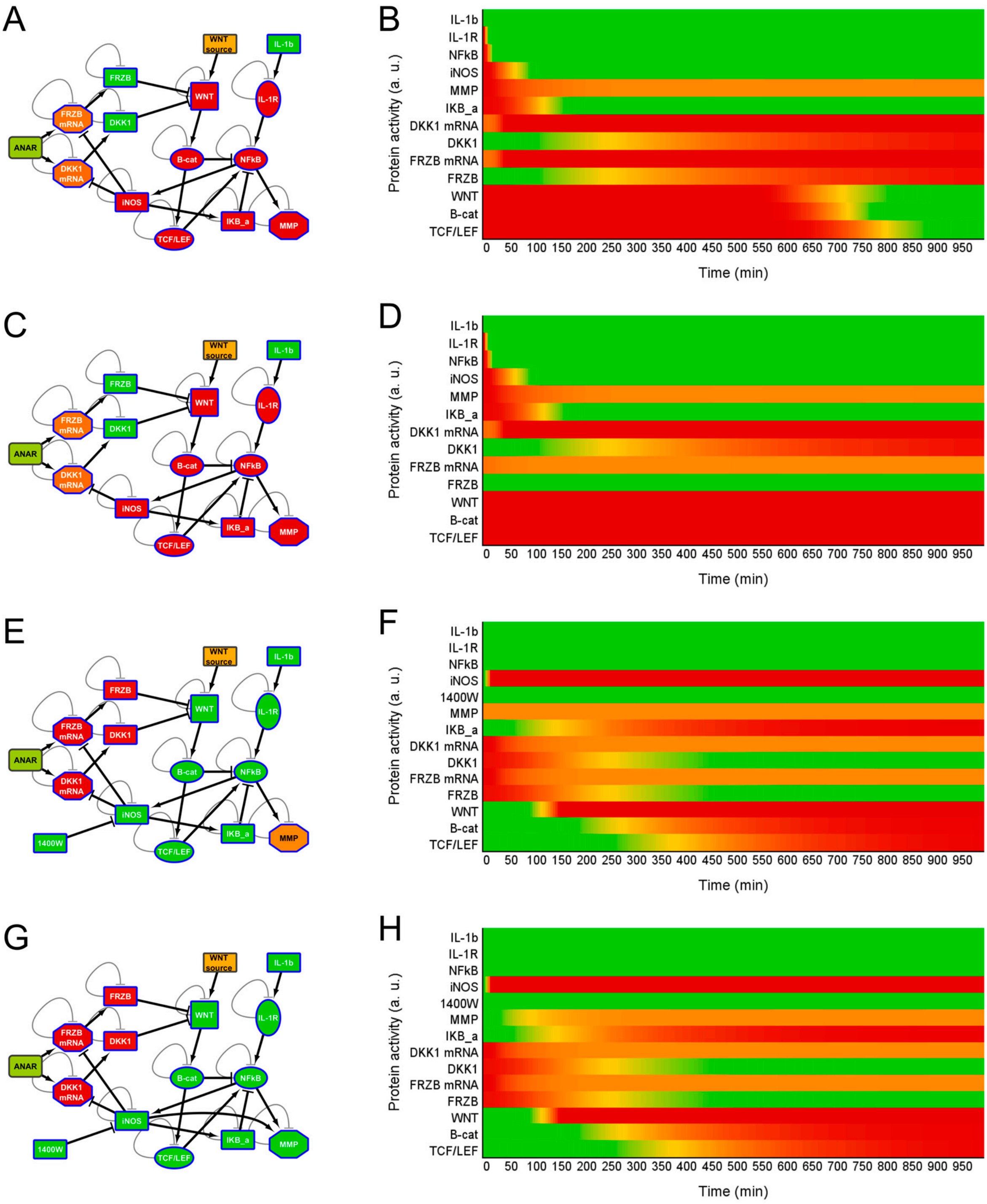

Supplemental Figure S5. ANIMO models used to test the hypothesis that IL1β can activate WNT signaling by downregulating DKK1 and FRZB via iNOS. A, B. Model 1, in which iNOS downregulates both DKK1 and FRZB resulting in upregulation of WNT activity; C, D. Model 2, the WNT and IL pathways including the WNT antagonists DKK1 and FRZB. When only DKK1 is inhibited by iNOS, as is described [27] there is no WNT activity; E,F. Model 3, 1400W is added to inhibit iNOS, thereby restoring DKK1 and FRZB expression resulting in inhibition of WNT activity; G,H. Model 4, iNOS regulates MMP expression. If 1400W is added the expression of MMP is also decreased.

Step 5: Adaptation of the model to fit the biological data

In our ANIMO models of the WNT and IL1β pathways in chondrocytes we used very simple reaction kinetics, in which activation via post-translational modification was considered a fast reaction, and activation via gene expression was considered a slow reaction. This simplification was

sufficient to describe the trends of the activation, but not the in a realistic time line. We have therefore changed the time scale of the model to match the timing of events as reported by the experimental data. This was done achieved by comparing our experimental data with timing information (Figures 3E, 3F, and Figure 4D) to the time scale of the model. As experimental data showed events that are about 4 times slower than the model interactions, we lowered the *k* parameter of each interaction by 4-fold for all interactions in the ANIMO model. In addition, we observed in our experimental data that FRZB was inhibited at a slower rate than DKK1. In our model, we lowered the value of the *k* parameter for the iNOS – ┤ FRZB mRNA interaction to 0.001 and the FRZB mRNA to FRZB protein to 0.001 to match this better.

Table S2. Parameter settings for models in figures S4-S5. To simplify the model construction, the k-values used in the models presented in this article were mostly chosen among ANIMO's qualitative range, which has a direct correspondence with numerical values as follows: very slow = 0.001, slow = 0.002, medium = 0.004, fast = 0.008, very fast = 0.016.

**Model figure S2A**	
Interaction	k-values
activation	
IL1b --> IL1R	0.016
IL1R --> NFkb	0.016
NFkb --> IL1b	0.001
NFkb --> iNOS	0.002
iNOS --> IKb_a	0.001
inhibition	
IL1b --l IL1b	4.40E-04
IL1R --l IL1R	4.40E-04
NFkb --l NFkb	0.01
IKb_a --l NFkb	0.008
Model Figure S2B	
activation	
bcat-mRNA --> bcat	0.008
bcat --> TCF/LEF	0.016
inhibition	
Frizzled --l GSK3b	0.016
LRP --l GSK3b	0.016
GSK3b --l bcat	0.016
GSK3b --l Axin	0.016
GSK3b --l APC	0.016
Model figure S2C	
activation	
bcat-mRNA --> bcat	0.008
WNT --> LRP	0.016
WNT --> Frizzled	0.016
bcat --> TCF/LEF	0.016
inhibition	
GSK3b --l APC	0.016
GSK3b --l Axin	0.016
GSK3b --l bcat	0.016

LRP --| GSK3b	0.016
Frizzled --| GSK3b	0.016
Model figure S2D	
WNT --> bcat	0.003
bcat --> TCF/LEF	0.016
Models 1- 4 (figure S3A-D)	
Interaction	k-values
activation	
ANAR --> DKK1 mRNA	0.002
ANAR --> FRZB mRNA	0.002
B-cat --> TCF/LEF	0.002
DKK1 mRNA --> DKK1	0.002
FRZB mRNA --> FRZB	0.002
IL-1R --> NFkB	0.016
IL-1b --> IL-1R	0.016
NFkB --> MMP	0.001
NFkB --> iNOS	0.002
TCF/LEF --> NFkB	0.001
WNT --> B-cat	0.003
WNT source --> WNT	0.016
iNOS --> IKB_a	0.002
iNOS --> MMP	0.002
inhibition	
1400W --| iNOS	0.016
B-cat --| B-cat	0.001
B-cat --| NFkB	0.004
DKK1 --| DKK1	6.00E-04
DKK1 --| WNT	0.016
DKK1 mRNA --| DKK1 mRNA	0.004
FRZB --| FRZB	6.00E-04
FRZB --| WNT	0.016
FRZB mRNA --| FRZB mRNA	0.004
IKB_a --| IKB_a	0.001
IKB_a --| NFkB	0.002
IL-1R --| IL-1R	0.002
MMP --| MMP	0.003
NFkB --| NFkB	0.002
TCF/LEF --| TCF/LEF	0.002
WNT --| WNT	0.004
iNOS --| DKK1 mRNA	0.016
iNOS --| FRZB mRNA	0.016
iNOS --| iNOS	1.00E-04

Fig 2B

Node name	Initial activity
ANAR	67
B-cat	0
DKK1	100
DKK1 mRNA	26
FRZB	100
FRZB mRNA	28
IKB_a	3
IL-1R	2
IL-1b	100
MMP	0
NFkB	0
TCF/LEF	0
WNT	0
WNT source	42
iNOS	100

Fig S4B

Node name	Initial activity
IKB_a	0
IL-1R	0
IL-1b	100
NFkB	0
iNOS	0

Fig S4D

Node name	Initial activity
APC	20
Axin	40
Frizzled	0
GSK3b	100
LRP	0
TCF/LEF	0
bcat	0
bcat-mRNA	50

Fig S4F	
Node name	Initial activity
APC	20
Axin	40
Frizzled	0
GSK3b	100
LRP	0
TCF/LEF	0
WNT	100
bcat	0
bcat-mRNA	50

Fig S4H	
Node name	Initial activity
TCF/LEF	0
WNT	100
bcat	0

Fig S4J	
Node name	Initial activity
IKB_a	0
IL-1R	0
IL-1b	100
NFkB	0
TCF/LEF	0
WNT	100
bcat	0
bcat-mRNA	50
iNOS	0

Fig S5A	
Node name	Initial activity
ANAR	67
B-cat	0
DKK1	100
DKK1 mRNA	26

FRZB	100
FRZB mRNA	28
IKB_a	3
IL-1R	2
IL-1b	100
MMP	0
NFkB	0
TCF/LEF	0
WNT	0
WNT source	42
iNOS	0

Fig S5C

Node name	Initial activity
ANAR	67
B-cat	0
DKK1	100
DKK1 mRNA	26
FRZB	100
FRZB mRNA	28
IKB_a	3
IL-1R	2
IL-1b	100
MMP	0
NFkB	0
TCF/LEF	0
WNT	0
WNT source	42

Fig S5E

Node name	Initial activity
1400W	100
ANAR	67
B-cat	100
DKK1	5

DKK1 mRNA	0
FRZB	5
FRZB mRNA	0
IKB_a	100
IL-1R	100
IL-1b	100
MMP	33
NFkB	100
TCF/LEF	93
WNT	100
WNT source	42
iNOS	100

Fig S5G

Node name	Initial activity
1400W	100
ANAR	67
B-cat	100
DKK1	5
DKK1 mRNA	0
FRZB	5
FRZB mRNA	0
IKB_a	100
IL-1R	100
IL-1b	100
MMP	100
NFkB	100
TCF/LEF	93
WNT	100
WNT source	42
iNOS	100
